# Taguchi orthogonal optimization for the oxidative degradation of 2-chlorophenol using zero valent iron-activated persulfate†

**DOI:** 10.1039/d5ra01495f

**Published:** 2025-07-15

**Authors:** Nikka Mae Macalde Buenaobra, Chenju Liang, Xuyen Thi Hong Luong, Khyle Glainmer Nagtalon Quiton

**Affiliations:** a Department of Environmental Engineering, National Chung Hsing University 145 Xingda Road, South Dist. Taichung City 402202 Taiwan cliang@nchu.edu.tw nmmbuenaobra@mymail.mapua.edu.ph xuyenluong@yahoo.com +886-4-22856610 +886-4-22856610; b School of Chemical, Biological, and Materials Engineering and Sciences, Mapúa University 658 Muralla St., Intramuros Manila 1002 Philippines kgnquiton@mapua.edu.ph

## Abstract

Chlorophenols (CPs) are persistent toxicants and major groundwater pollutants due to their carcinogenic properties. This study focused on optimizing the degradation of 2-chlorophenol (2-CP) in the aqueous phase using zero-valent iron (ZVI)-activated sodium persulfate (SPS). The Taguchi Design of Experiment methodology (L9 orthogonal array) and ANOVA statistical analysis were applied to identify optimal conditions, with key parameters including initial pH, SPS concentration, and ZVI concentration. Results indicated that SPS concentration had the most significant impact on 2-CP degradation. Confirmation tests conducted at the optimal conditions (pH = 6, SPS = 60 mM, ZVI = 60 mM) achieved 100% 2-CP degradation within 10 min, characterized by rapid 2-CP degradation, SPS decomposition, and 2-CP mineralization. Experiments with 2-CP spiked reverse osmosis water and groundwater samples showed total organic carbon removal rates of 75% and 67%, respectively, with discussions highlighting the potential effects of groundwater constituents on degradation efficiency. ZVI surface analysis through SEM-EDS identified iron oxide crystal formation on recovered iron particles, while XRD analysis confirmed the presence of Fe_3_O_4_ on the particle surface post-reaction. These findings underscore the effectiveness of ZVI-activated SPS as a promising approach for 2-CP degradation in natural groundwater systems, contributing to supportable groundwater remediation efforts.

## Abstract 2

Zero-valent iron-activated persulfate: a promising approach for 2-chlorophenol degradation in groundwater.

## Graphical abstract

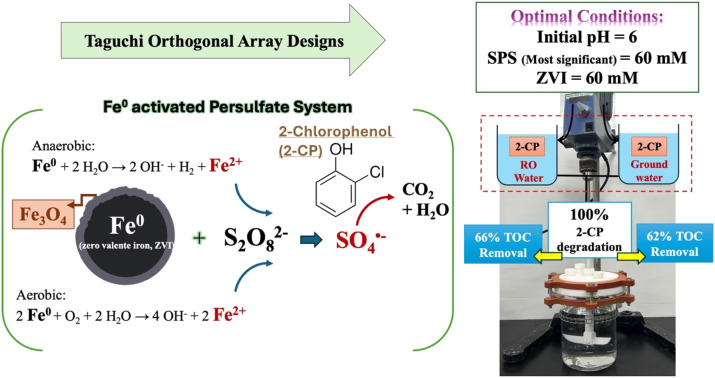

## 1. Introduction

Water security is an international priority such that various methods are being pursued to overcome challenges in water quality globally. About one-third of all freshwater withdrawals worldwide come from groundwater, which is crucial for industrial, agricultural, and residential use.^1^ Groundwater contamination can occur due to surface activities such as industrial spills or the release of stored waste. This contamination degrades soil and water quality, adversely impacting soil microorganisms, animals, and human health.^2,3^ Approximately 80% of industrial wastewater is left untreated and discharged back into the environment according the United Nation Water Development Report.^4^ Moreover, untreated industrial effluents from industries like paint, pesticide, and petrochemicals contribute to contamination in soil and groundwater. Chlorophenols (CPs), toxic byproducts of water chlorination, are persistent pollutants with carcinogenic properties that adversely affect the human nervous and respiratory systems.^5^ CPs are classified as toxic pollutants regulated and monitored under the Philippines' water quality guidelines. Contamination by chlorophenols, such as 2-chlorophenol (2-CP), often originates from industrial effluents, including those from glue manufacturing and the textile industry.^6^ 2-CP is also used as insecticides and disinfectant agents.^7^ Three types of chlorophenols (*i.e.*, 2-chlorophenol, 2,4-dichlorophenol, and 2,4,6-trichlorophenol) are listed as secondary parameters-organic contaminants and limited within a range of concentration per water according to the Philippine water quality guidelines. The permissible limits for phenol and phenolic substances across various classifications range in groundwater from less than 0.001 mg L^−1^ to a maximum of 0.05 mg L^−1^ (see Table S1, ESI†).^6^ Specifically, conventional subsurface contamination remediation method, pump-and-treat approach, typically rely on *ex situ* treatments such as adsorption, leading to secondary waste issues from spent adsorbents, or biological treatments, which often require prolonged treatment times and are sensitive to operational conditions. These limitations underscore the need for a more effective *in situ* remediation strategy that enables rapid and direct destruction of contaminants without generating additional treatment challenges.

Advanced oxidation processes (AOPs) pertain to methods that involve the application of hydroxyl radicals (HO˙) as the species to destroy organic compounds into less harmful substances.^8^*In situ* chemical oxidation (ISCO) remediation technique is one of AOP applications used to oxidize subsurface contaminants such as chlorinated organic compounds and petroleum hydrocarbons.^9–12^ The persulfate (S_2_O_8_^2−^, PS) oxidant relies on its excellent persistence within soil matrix and the formation of reactive radical species such as sulfate radicals (SO_4_˙^−^). Hence, ISCO oxidant sodium persulfate (SPS), a type of persulfate salt, has been studied for its sustained activity in aquifer materials and its effectiveness in oxidizing various contaminants, similar to the oxidative reactions of Fenton's reagent with hydrogen peroxide (H_2_O_2_).^13^ In *in situ* chemical oxidation applications that adopt advanced oxidation processes for subsurface remediation, persulfate is often preferred over hydrogen peroxide as a source of sulfate and hydroxyl radicals due to its greater stability in subsurface soil environments and natural aqueous media. Its persistence allows for more effective transport and diffusion through soil pores, thereby enhancing contact with contaminated zones. Once introduced into the subsurface, persulfate can be activated to generate sulfate radicals through various methods, including heat, transition metals, ultraviolet (UV) light, and alkaline conditions. However, heat activation demands high energy input, UV light cannot effectively penetrate soil particles, and significant amounts of alkaline agents are needed to overcome soil buffering capacity. In contrast, transition metals such as iron can rapidly activate persulfate under ambient conditions, efficiently generating sulfate radicals to oxidize dissolved contaminants. Zero valent iron (Fe^0^, ZVI) is a strong reductant (eqn (1)) and has been used as a source of ferrous ion (Fe^2+^) (eqn (2)–(4)) to activate PS (eqn (5)) for generating SO_4_˙^−^.^14^ Metal–organic frameworks (MOFs) and their advanced iron-based composites have exhibited potential as persulfate activators, offering a possible improvement in activation efficiency. The underlying principles and related mechanisms are discussed in the review by Zhang *et al.* (2025).^15^ The overall reaction between ZVI and PS is shown as eqn (6), with a stoichiometric S_2_O_8_^2−^/Fe^0^ molar ratio of 1.5. Additionally, the overall reaction between Fe^2+^ and PS by combing eqn (5) and (7) occurs at a stoichiometric molar ratio Fe^2+^/S_2_O_8_^2−^ of 2 (eqn (8)). The equations are quoted from the compilation of Liang and Guo (2010).^15^

1 Fe^0^ → Fe^2+^ + 2e^−^*E*^0^ = 0.447 V

2 Aerobic condition: 2Fe^0^ + O_2_ + 2H_2_O → 2Fe^2+^ + 4OH^−^

3 Anaerobic condition: Fe^0^ + 2H_2_O → Fe^2+^ + 2OH^−^ + H_2_

4 Interaction between ZVI and PS: Fe^0^ + S_2_O_8_^2−^ → Fe^2+^ + 2SO_4_^2−^

5 Fe^2+^ + S_2_O_8_^2−^ → Fe^3+^ + SO_4_˙^−^ + SO_4_^2−^

6 Fe^0^ + 1.5S_2_O_8_^2−^ → Fe^3+^ + 3SO_4_^2−^

7 SO_4_˙^−^ + Fe^2+^ → Fe^3+^ + SO_4_^2−^*k* = 4.6 × 10^9^ M^−1^ s^−1^

8 S_2_O_8_^2−^ + 2Fe^2+^ → 2SO_4_^2−^ + 2Fe^3+^

SPS activated by ZVI and Fe^2+^ have been demonstrated effectively to degrade 2,4,6-trichlorophenol in aqueous solution.^16^ Upon reaction, the corrosion of ZVI led to the formation of magnetite (Fe_3_O_4_) or maghemite (γ-Fe_2_O_3_).^16^ Degradation of 2-CP through UV-assisted Fenton oxidation has also been studied with an initial concentration of 50 to 100 mg L^−1^, which is in the range of the reported effluent concentration range (*e.g.*, 0.1 to 1600 mg L^−1^). For example, the degradation of 2-CP, with initial concentration of 50 mg L^−1^ and 18 mg L^−1^ of Fe at pH 3, resulted in 82.7% with 0.2% TOC removal.^17^ Moreover, UV-assisted TiO_2_ exhibited 24% of 2-CP degradation, while 34.4% and 74% of 2-CP degradations using photolytic ozonation with initial pHs 6 and 9 were observed, respectively.^18–21^ Common intermediates observed during the course of 2-CP degradation include chlorobenzoquinone, chlorohydroquinone, phenol, catechol, hydroxyhydroquinone, maleic acid, oxalic acid, benzoquinone, and hydroquinone.^18,22–26^ 2-CP can be degraded through AOP; however, the oxidation process seemed not to achieve complete 2-CP degradation or mineralization.

In this study, the SPS/ZVI system to degrade 2-CP was investigated through batch experiments and would aid as an alternative in further applications. The scope of the present study included optimization of the process parameters on the degradation of 2-CP under ZVI activated persulfate AOP process using the Taguchi Design of Experiment methodology. Specifically, it focused on determining the effect of initial pH, SPS concentration, and ZVI concentration. Moreover, the study also included evaluation of 2-CP degradation and mineralization in spiked reagent water and field groundwater samples.

## 2. Methodology

### 2.1. Chemicals and materials

All the chemicals used in the study were of analytical grade and utilized without further purification. Water was prepared using a reverse osmosis (RO) purification system (Sky Water XL-300A). 2-Chlorophenol and potassium hydrogen phthalate (KHP) were purchased from Thermo Fisher Scientific (Belgium). Sodium persulfate was purchased from San Yuan Chemicals (Taiwan). Nitric acid (HNO_3_), ethanol, acetic acid (CH_3_COOH), phosphoric acid (H_3_PO_4_), and sulfuric acid (H_2_SO_4_) were purchased from J.T. Baker (USA). Acetonitrile (C_2_H_3_N) is purchased from Aencore Chemicals (Australia). Sodium hydroxide (NaOH) and magnesium chloride (MgCl_2_) were purchased from Sigma-Aldrich (USA). Sodium chloride (NaCl) and potassium chloride (KCl) were purchased from Honeywell (Germany). Calcium sulfate dihydrate (CaSO_4_·2H_2_O) was purchased from Merck (Germany). Zero valent iron (Fe^0^) was purchased from Alfa Aesar (USA). Potassium iodide (KI) and sodium bicarbonate (NaHCO_3_) were purchased from Union Chemical Works (Taiwan). Mixed anion standard solution was purchased from CPA Chem (Bulgaria). FerroVer Iron Reagent and Ferrous Iron Reagent were purchased from Hach Company (USA).

### 2.2 Experimental procedure

ZVI particles (40 g) were prewashed with HNO_3_ (250 mL of 0.01 M solution) in a 250 mL storage bottle to be agitated using a reciprocal shaker at 300 rpm for 30 min.^27^ Thereafter, the acid was poured out and replaced with degassed RO water for rinsing^28^ (see Fig. S1(a), ESI†). This rinsing process was repeated with RO water until the measured pH approached neutrality, in which water visibly becomes clearer each rinsing (Fig. S1(b), ESI†). The washed ZVI particles underwent nitrogen purging for drying, and the nitrogen atmosphere was maintained to preserve the ZVI particles.^29^

An initial 2-CP concentration of 1 mM (128.6 mg L^−1^) was prepared for each experiment. The experimental procedure followed in this study closely follows that reported by Liang and Lai.^29^ A schematic of the setup is provided in Fig. S2(a)† (ESI†), and a corresponding photograph of the actual apparatus is shown in Fig. S2(b) (ESI†). The reaction was conducted for 1 h and sampling took place every 10 min. At each designated sampling period, aqueous solution was withdrawn using a pipet through a port on the top-cover and filled into 40 mL brown borosilicate bottle with Teflon-lined caps, in which 0.25 mL of ethanol as a radical scavenging agent was added prior to sampling.^14^ Each filled brown bottle was agitated after sampling, placed on a reciprocal shaker (DLAB, SK-L330-Pro) at 300 rpm for 1 min to ensure well mixing of the quencher and then stored at 4 °C for analysis. The pH and oxidation–reduction potential (ORP) were also measured for each sample using a dual channel benchtop meter (Hanna, HI5222). Three control tests were performed with 2-CP in water alone, 2-CP and SPS at SPS concentration of 300 mM, and 2-CP at ZVI concentration of 500 mM.

The Taguchi orthogonal method for obtaining optimized experimental design was proposed for the investigation of the influence of variation in different parameters.^30^ This method was selected for its efficiency in significantly reducing the number of experimental runs required compared to a full factorial design, especially when exploring multiple factors at several levels. In this study, with three factors (ZVI concentration (mM), SPS concentration (mM), and initial pH), each investigated at three levels, a full factorial design would necessitate 27 experiments. To efficiently identify the most influential parameters and their optimal operations in this investigation, the Taguchi L9 orthogonal array was chosen, requiring only 9 experimental runs. This approach allowed for a comprehensive analysis of the main effects of these factors under a fixed 2-CP concentration at 1 mM, while optimizing experimental resources and time. The Taguchi L9 experimental design, detailing the specific levels for each factor, is presented in Table 1. Under each specified experimental condition, the 2-CP degradation percentage was calculated. It is noted that the SO_4_˙^−^ half reaction (eqn (9)) combined the oxidation half reaction of 2-CP (eqn (10)) is shown in eqn (11). It is shown that 26 moles of SO_4_˙^−^ are required to oxidize 1 mole of 2-CP. According to the 1 : 1 molar ratio of SO_4_˙^−^ generated by Fe^2+^ activated PS (eqn (5)). 26 moles of the persulfate anions are stoichiometrically required to oxidize 1 mole of 2-CP.

9 SO_4_˙^−^ + e^−^ → SO_4_^2−^*E*^0^ ≈ 2.6 V

10 C_6_H_4_ClOH + 11H_2_O → 6CO_2_ + 27H^+^ + Cl^−^ + 26e^−^

11 26SO_4_˙^−^ + C_6_H_4_ClOH + 11H_2_O → 6CO_2_ + 27H^+^ + Cl^−^ + 26SO_4_^2−^

**Table 1:** Experimental design of Taguchi L9 orthogonal array

Items		Factors			
		Initial pH	SPS (mM)	ZVI (mM)	2-CP (mM)
Level	1	2	15	30	
	2	6	30	60	1.0
	3	10	60	120	
Experiment	1	2	15	30	1.0
	2	2	30	60	
	3	2	60	120	
	4	6	15	60	
	5	6	30	120	
	6	6	60	30	
	7	10	15	120	
	8	10	30	30	
	9	10	60	60	

The level of SPS concentration was determined based on the molar ratio of 26 : 1, such that range of concentrations of 15 mM (3.57 g L^−1^), 30 mM (7.14 g L^−1^), and 60 mM (14.28 g L^−1^) were set to evaluate effects of shortness to excess of SPS in the system. Additionally, ZVI concentrations of 30 mM (1.68 g L^−1^), 60 mM (3.35 g L^−1^), and 120 mM (6.7 g L^−1^) were used in correlation to the molar ratio of SPS to Fe^2+^ (1 : 2) (eqn (8)). The initial pH values are based on acidic, neutral, and basic conditions. With 9 sets of experiments, it was aimed to use the generated experimental design for exploring the effects of each parameter in the degradation of 2-CP.

The Taguchi orthogonal array produces the signal-to-noise (S/N) ratio to assess the impacts of response variables, with the S/N ratio measured in decibels (dB). Higher S/N ratios indicate greater efficiency in 2-CP removal. Raw data was transformed into S/N ratios using eqn (12).12
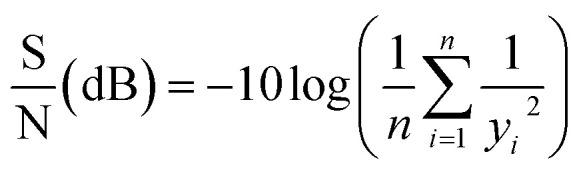
where *n* is the number of experiments, and *y*_*i*_ is the 2-CP degradation percentage of each experiment. Furthermore, the contribution percentage of each parameter was determined using Analysis of Variance (ANOVA).^30^ The contribution of each factor in the degradation process was calculated using eqn (13).13
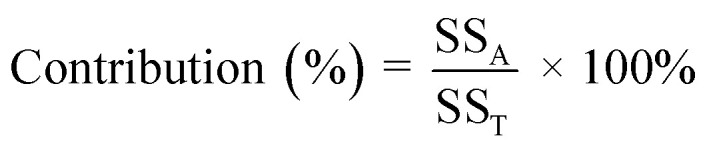
where SS_A_ is the sum of square of factor (*e.g.*, factor A) and SS_T_ is the total sum of squares.

### 2.3 Analytical methods

2-CP was analyzed using high performance liquid chromatography equipped with photo diode array detector (HPLC/PDA) (PerkinElmer/Flexar LC system) equipped with a Brownlee SPP C18 column (PerkinElmer, 4.6 × 150 mm, 2.7 μm) at a wavelength of 275 nm. The mobile phases are C_2_H_3_N and 1.5% CH_3_COOH in water (v/v = 45 : 55) at a flow rate of 0.4 mL min^−1^, run time of 7 min,^31,32^ and a retention time of approximately 5.5 min (as shown in Fig. S3 (ESI†), a 3D spectrum used to identify retention time and wavelength for 2-CP) (limit of quantification (LOQ) = 0.5 mg L^−1^). SPS was standardized using a spectrophotometry method measured at a wavelength of 400 nm (T6U, Persee Analytics).^33^ A sample standard curve for SPS quantification is shown in Fig. S4 (ESI†) (LOQ = 5.94 mg L^−1^). The ZVI was characterized using Brunauer–Emmett–Teller surface area analyzer (BET) (Porous Materials, Inc. C-BET-201A). Field emission scanning electron microscope (JSM-6700F, Jeol) and partnered with energy dispersive X-ray spectroscopy (EDS) spectrophotometer (INCA x-sight, Oxford Instruments) was used to characterize iron surface. Moreover, an X-ray diffractometer (XRD) (Empyrean, Malvern Panalytical) was used to characterize iron and generate an XRD gonio scan with a step size of 0.0670° 2*θ*, a scan step time of 9.95 s, a step size ranging from 5° 2*θ* to 90° 2*θ*, at 40 mA and 45 kV.

Total iron and ferrous ion were calibrated using iron reagents, FerroVer reagent (Hach), in accordance with the 1,10-phenanthroline using a visible spectrophotometer method (T6U, Persee Analytics) at 510 nm ^34^ (LOQ = 0.01 mg L^−1^). To analyze the mineralization end-product Cl^−^, a Cl^−^ probe (Orion, Thermo Scientific) was used (LOQ = 1.0 mg L^−1^). An ionic strength adjuster (Orion 940011, Thermo Scientific) was used for standardization and measurement. A sample standard curve for chloride quantification can be seen in Fig. S5 (ESI†). Chloride ion quantification in groundwater was standardized and analyzed using ion chromatography (Metrohm 790) with eluents of deionized water, H_2_SO_4_, and NaHCO_3_. Mixed anion standard solution containing 1000 ppm of 7 components (*i.e.*, fluorides, chlorides, nitrites, bromides, nitrates, sulphates, and phosphates, CPA Chem) in water was used for standard stock solution for Cl^−^ quantification. Moreover, mixed cation standard solution containing 100 mg L^−1^ of mixed cations was synthesized and used to quantify common cations (*i.e.*, sodium using NaCl, potassium using KCl, calcium using CaSO_4_·2H_2_O, and magnesium using MgCl_2_), the chromatograph for cations as shown in Fig. S6 (ESI†). In order to analyze the mineralization of 2-CP during the course of the oxidation process, the total organic carbon (TOC) in the solution was also analyzed (OI Analytical Aurora 1030 TOC Analyzer). Analysis of TOC uses the principle of non-dispersive infrared detector to measure CO_2_.^35^

## 3. Results and discussion

### 3.1. Optimization using Taguchi orthogonal method

The BET analysis of ZVI particles revealed a particle diameter of 75 μm, a specific surface area of 0.4101 m^2^ g^−1^, and a median pore diameter of 94.96 Angstrom based on the 50th percentile point on the surface area.^36^ First, control tests were conducted with 2-CP in water alone, 2-CP with SPS, and 2-CP with ZVI. 2-CP in water was tested to evaluate the activity of hydrolysis to 2-CP degradation. Hydrolysis has been used to treat soil and groundwater contaminants with the aid of basic pH conditions or also called alkaline hydrolysis. A study by Huang *et al.*^37^ demonstrated that the addition of alkali can effectively hydrolyze certain types of chlorinated alkanes. In this study, as shown in Fig. S7 (ESI†), results show that 2-CP in water alone resulted in only 4% of 2-CP degradation after 1 h of agitation. Addition of 300 mM of SPS was then tested, which resulted in 16.7% 2-CP degradation while addition of 500 mM of ZVI resulted in 11.3% 2-CP degradation. The negligible degradation of 2-CP in water alone highlights resistance of 2-CP to natural breakdown processes. SPS improved degradation, indicating the role of persulfate as an oxidizing agent in breaking down 2-CP. However, the moderate degradation efficiency suggests that SPS activation is necessary to maximize its oxidative potential. The relatively lower efficiency compared to SPS alone suggests that ZVI's potential is limited without additional oxidants or synergistic reactions.

**Fig. 1:**
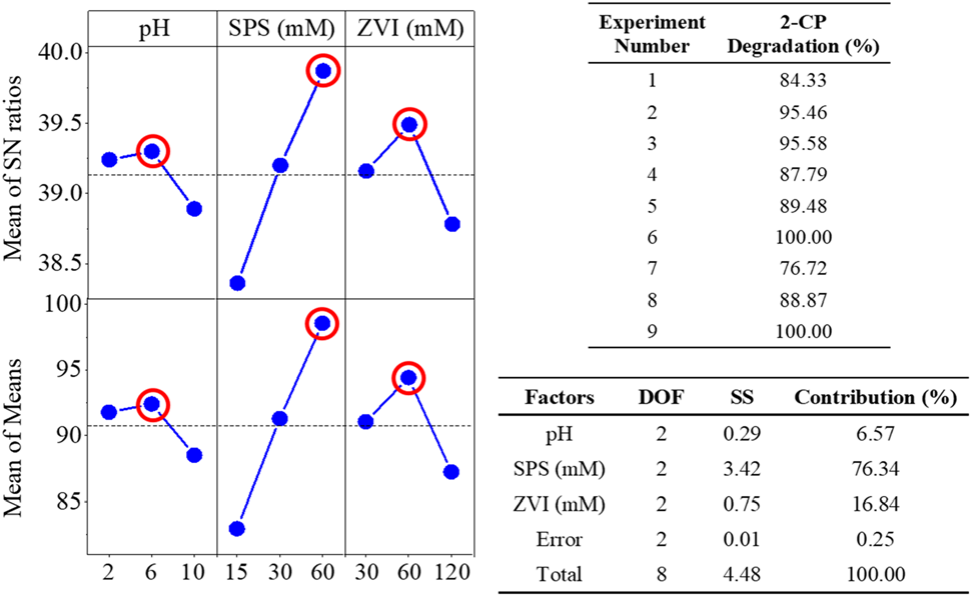
Main effects plot for S/N ratios and 2-CP degradation percentage means, ANOVA results, and 2-CP degradation percentage results.

The degradation of 2-CP using SPS/ZVI system was evaluated using Taguchi orthogonal array and the results of 2-CP degradation are shown in Fig. 1. With different levels under nine experimental conditions, along with the resulting S/N ratio values (data tabulated in Table S2 (ESI†)), ANOVA results are also presented. The highest 2-CP degradation (100.0%) and the highest S/N ratio (40.00 dB) were achieved in Experiments 6 and 9, which proves that 2-CP can be degraded with the use of SPS/ZVI system. On the other hand, the lowest 2-CP degradation (76.72%) corresponded to the lowest S/N ratio (37.7 dB) in Experiment 7.

In this study, the The-Larger-The-Best criterion was applied to assess the degradation of 2-CP in the SPS/ZVI system. As the delta value of the S/N ratio increases, a corresponding increase in 2-CP degradation is expected. This method involved ranking delta values to determine the significance of each factor, where a higher delta value suggests a stronger influence on the overall experimental results. Table S2 (ESI†) presents the response data for both means and S/N ratios and shows how each factor is ranked based on its impact on these outcomes. Fig. 1 illustrates the S/N ratio and average 2-CP degradation in relation to different factor levels. The results indicate that the highest S/N ratios and 2-CP degradation occur at level 2 for initial pH values and ZVI concentrations, while the highest value for SPS concentration is at level 3. Hence, the optimal operating conditions are initial pH value of 6 (level 2), ZVI concentration at 60 mM (level 2), and SPS concentration at 60 mM (level 3).

The experimental design was based on the Taguchi L9 orthogonal array, which evaluated three key factors, initial pH, SPS concentration, and ZVI concentration, each at three levels. This design was selected to efficiently screen and assess the influence of multiple parameters on 2-CP degradation using only nine experimental runs, rather than conducting a full factorial design which would require 27 experiments. However, a limitation of the Taguchi method is that the design does not allow for direct isolation of the individual effect of one factor (*e.g.*, ZVI concentration) on the response (*i.e.*, degradation efficiency) without considering the confounding influence of other varying factors across the orthogonal array. Therefore, while ZVI concentration certainly influences 2-CP degradation, the degradation efficiency observed in each experiment is a result of the combined effect of all three parameters under the specific conditions tested. The degradation efficiency of 2-CP after 1 h of reaction was used as the primary output, and these results are presented in the inserted table in Fig. 1. Although a more direct cause-effect relationship between ZVI concentration and degradation efficiency cannot be definitively concluded from this experimental design alone, the Taguchi analysis does provide insight into the relative importance of each factor, as discussed in the results and confirmed through the analysis of means and signal-to-noise ratio evaluations.

To evaluate the statistical significance of each factor on the degradation of 2-CP, a Pareto chart of standardized effects was generated at a 95% confidence level (Fig. S8, ESI†). The red vertical line represents the critical *t*-value of 2.571. Among the three factors, SPS concentration, ZVI dosage, and initial pH, only the SPS concentration exceeds this threshold, indicating that it has a statistically significant effect on the degradation process. This result highlights SPS as the dominant contributor to 2-CP degradation within the experimental design, aligning with the ANOVA results, which showed SPS contributing 76.3% of the observed effect. The ZVI and pH factors, while still influential to some degree (contributing 16.8% and 6.6%, respectively), fall below the critical value and are therefore not statistically significant within the tested range. The pronounced effect of SPS can be attributed to its role as the primary oxidant, generating sulfate radicals that initiate the degradation process. The strong dependence on SPS also suggests that increasing the oxidant concentration enhances the availability of reactive species, which accelerates contaminant breakdown. Meanwhile, although ZVI serves as an activator and pH influences the speciation and stability of radicals, their effects are comparatively limited in this experimental setup. This is also visible in the 2-CP degradation results in Fig. 1 that the highest results with complete or 95.58% 2-CP degradation and (Experiment 3, 6, and 9) resulted from systems with 60 mM SPS concentration.

To evaluate the effect of each factor on each other, an interaction plot was generated. An interaction plot helps to visualize and interpret how multiple factors interact in an experimental setup. As shown in Fig. S9 (ESI†) interaction plot of different factors, if the lines on the plot are not parallel and intersect, it indicates that there is an interaction between the factors, meaning the impact of one factor varies depending on the level of another factor. When lines cross each other, it suggests a strong interaction, and the extent of the crossing indicates how significant this interaction is. When SPS and ZVI are evaluated for their influences on each other, the lines are not exactly parallel but minimally crossing or touching, indicating that there is little significant interaction between the two factors. On the other hand, initial pH values are shown to have crossing lines in both SPS and ZVI concentrations, indicating that the factor of initial pH values has weighty interaction on the other factors.

A confirmation test was performed to determine the efficiency of the optimal conditions acquired from the Taguchi experiments. A linear regression analysis was carried out to model the degradation of 2-CP using the SPS/ZVI system. The model was developed using the ordinary least squares method that reduces the sum of the squared differences between the observed and predicted values. Consequently, the complete model is shown in eqn (14):^38^

14 2-CP degradation (%) = 85.49 − 0.407*x*_1_ + 0.3313*x*_2_ − 0.0533*x*_3_

where *x*_1_ is the initial pH value, *x*_2_ is the SPS concentration (mM), and *x*_3_ is the ZVI concentration (mM). Moreover, a normal probability plot of residuals is generated to confirm that the residuals follow a normal distribution. The normal probability plot of the residuals indicates that most of the residuals are well-behaved, as they lie close to a straight line as shown in Fig. S10 (ESI†). Using the model, optimal conditions were predicted to result in complete degradation. Through a confirmation test such that same experimental procedure was employed together with the optimized factors, it was proved that complete degradation was achieved.

**Fig. 2:**
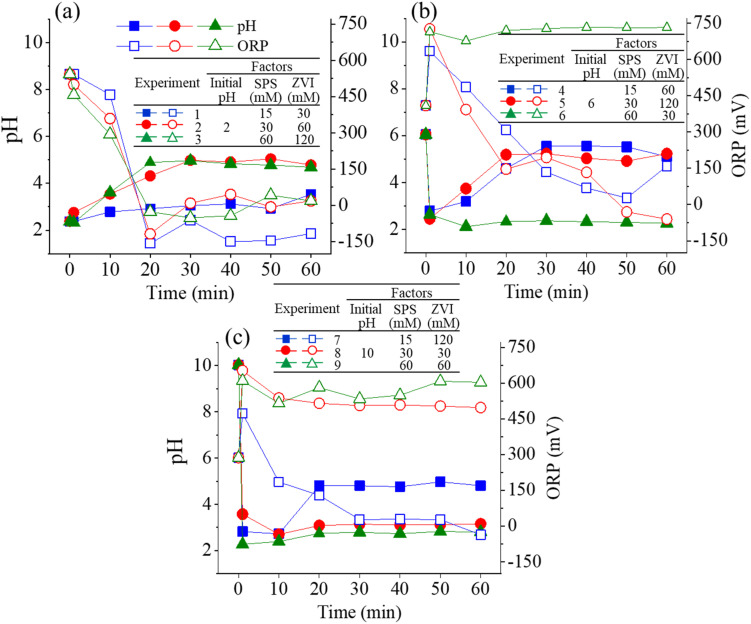
pH and ORP variations for Taguchi experiments at initial pH (a) 2, (b) 6, and (c) 10.

Fig. 2 illustrates the pH and ORP variation for Taguchi experiments divided from each initial pH value. As shown in Fig. 2(a), the molar ratio for the three experiments is the same at 1 : 2 (SPS/ZVI molar ratio), indicating an excess of iron in the reaction system compared to the theoretical stoichiometric ratio of 1 : 1.5 (SPS/ZVI molar ratio) as presented in eqn (6). Under this condition, persulfate would be completely consumed, resulting in a diminished oxidation reaction, with residual ZVI dominating the system. Consequently, when the oxidation reaction is predominant, the ORP increases, accompanied by an acidic environment enriched in H^+^ and SO_4_^2−^, as described in eqn (4). Both pH and ORP variations exhibited the same trends for the three experiments. pH value, which is initially 2, increased significantly at 20 min, and thereafter became steady until 60 min. Conversely, the ORP value, initially at approximately 550 mV, decreased significantly to a negative value at 20 min, in accordance with the increase of pH value, and became steady through the process. Then, shown in Fig. 2(b) with pH value of 6, Experiments 4 and 5 have a molar ratio of 1 : 4 (SPS/ZVI) while Experiment 6 have a molar ratio of 2 : 1 (SPS/ZVI). When SPS is in excess relative to iron (*i.e.*, the SPS/ZVI molar ratio exceeds the theoretical ratio of 1.5, as indicated in eqn (6)), the reaction system maintains an acidic pH and elevated ORP. However, despite the presence of an oxidizing environment, the ORP in the SPS/ZVI system may be higher than that observed in the SPS-only oxidation system. Experiments 4 and 5 have similar trends wherein initial pH value at 6 rapidly decreased to acidic conditions but increased significantly at 20 min, in which it became steady through the process. Experiment 6 with a higher SPS, showed the decrease of pH to acidic level. The decomposition of persulfate produces bisulfate (HSO_4_^−^) as a byproduct, which generally lowers the pH. Typically, the pH remains above 2 unless extremely high concentrations of persulfate are used. Once persulfate is nearly depleted, the pH increases rapidly in accordance with eqn (2) and (3), reflecting the natural reactions of ZVI under both aerobic and anaerobic conditions. Meanwhile, the ORP continues to decline as the ZVI reduction process (eqn (1)) begins to dominate the system. In the case of Experiment 6, it can be suggested that persulfate was not completely exhausted, which shows that the oxidation significantly governed the system through the process with ORP at approximately 750 mV. This is further supported by the ORP values. Experiments 4 and 5 exhibit ORP trends similar to those in Experiments 1 to 3, where negative ORP values were observed, indicating that ZVI governed the system *via* reductive reactions (eqn (1)) at certain time points. In contrast, Experiment 6 showed an initial increase in ORP within the first minute, which remained elevated throughout the process, suggesting that SPS continued to dominate the system through oxidative reactions. Correspondingly, Experiments 8 and 9, which used a 4 : 1 molar ratio of SPS to ZVI (indicating a higher SPS concentration), produced results similar to Experiment 6: the initial pH of 10 decreased and remained acidic throughout the process, while the ORP value stayed high. On the other hand, Experiment 7 with molar ratio of 1 : 1 (SPS/ZVI) resulted in similar results with molar ratios where ZVI has higher concentration such that initial decrease to acidic condition is met at 1 min, but eventually increased at 20 min, which remained constant through the process. Additionally, ORP value exhibited a significantly steady linear decrease until 30 min and did not reach a negative value until 60 min. Overall, the ORP variation over time across the nine experimental conditions reflects the dynamic interplay between oxidizing (SPS) and reducing (ZVI) species, primarily governed by the initial pH and SPS/ZVI molar ratio. Note that the stoichiometric SPS to ZVI molar ratio is 1.5, as indicated in eqn (6). At lower SPS/ZVI ratios (*e.g.*, 1 : 2 or 1 : 4), the system initially undergoes oxidation but quickly transitions to a reduction-dominated process, evidenced by significant ORP drops to negative values following persulfate depletion and pH recovery. In contrast, at higher SPS/ZVI ratios (*e.g.*, 2 : 1 or 4 : 1), the ORP remains elevated, and the pH stays acidic, indicating sustained oxidation and incomplete consumption of persulfate. Intermediate conditions (*e.g.*, 1 : 1 ratio) show a delayed transition, where oxidation governs early stages, followed by a gradual shift to reduction as the system evolves. These trends underscore the critical roles of pH and reactant balance in shaping redox behavior during the reaction process.

### 3.2. Application to 2-CP spiked field groundwater

**Fig. 3:**
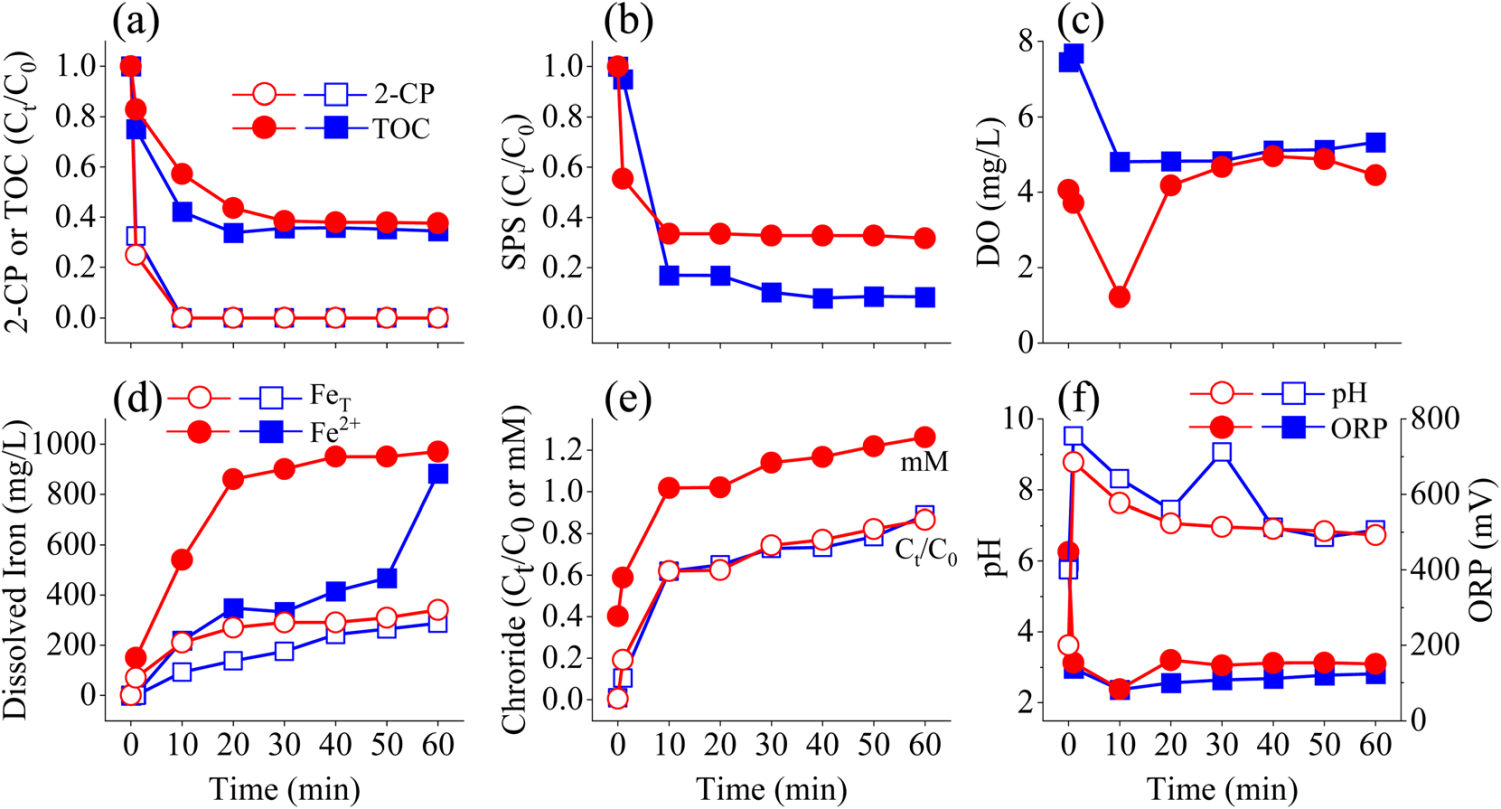
Results for (a) 2-CP degradation and TOC, (b) SPS, (c) DO, (d) dissolved iron, (e) chloride ion, and (f) pH and ORP variations in the system of 2-CP/SPS (14.28 g L^−1^)/ZVI (3.35 g L^−1^) in 2-CP spiked RO water and groundwater samples. Symbols: squares indicate RO water samples; circles indicate groundwater samples.

The ANOVA results suggest that optimizing SPS dosage is the most critical factor for improving treatment efficiency, while maintaining adequate ZVI levels and considering pH stability can further support system performance under field conditions. Optimized experimental conditions were applied in 2-CP spiked groundwater. Results on RO water and groundwater samples are discussed comparatively. Groundwater samples were sourced from a groundwater monitoring well site, which is located in the Department of Environmental Engineering of National Chung Hsing University, as shown in Fig. S11 (ESI†). The well is 15 meters deep with a diameter of 4 inches. Groundwater was collected and characterized as shown in Table S3 (ESI†). Whereas the RO water sample has an initial pH of 6.0, the groundwater sample begins at a similar, neutral pH. Moreover, the ORP value of RO water sample was approximately 400 mV while groundwater sample was lower at 168 mV. A lower ORP value can indicate a reductive environment, which can be caused by a lower dissolved oxygen (DO) at 4 mg L^−1^ in groundwater sample, as compared to 7 mg L^−1^ in RO water. In water sourced from wells, the depth can lead to lower DO value, which is also correlated to the reduction processes that can prevail, along with the presence of reducing materials. For example, elevated Ca^2+^ (70 mg L^−1^), Mg^2+^ (14 mg L^−1^), and hardness of 236.50 mg as CaCO_3_/L were measured. Consequently, 14 mg L^−1^ of Cl^−^ was measured as presented in Table S3 (ESI†). Conclusively, DO levels after 2-CP degradation in RO water and groundwater tests were 5.3 mg L^−1^ and 4.5 mg L^−1^, respectively, as shown in Fig. 3(c).

As shown in Fig. 3(a), rapid 2-CP degradation was observed in the groundwater sample, mirroring the RO water test results, and complete degradation was achieved in both water samples by 10 min of treatment. Additionally, the TOC removal efficiencies were 66% for the RO water test and 62% for the groundwater test. Contrastingly, mineralization was slightly lower in the groundwater test, suggesting that while the treatment process achieved complete 2-CP degradation, full mineralization was not attained. This could be possibly attributed to the interference of groundwater ions affecting the mineralization of 2-CP. As shown in eqn (11), complete transformation of 2-CP leads to CO_2_ as one of the ending products. As shown in Fig. 3(e), RO water test and groundwater test resulted in 89% and 87% of chloride transformation, respectively. This also shows that as complete mineralization was not complete, the entirety of chlorine in the structure of 2-CP was not completely transformed into an ion. Conclusively, 2-CP was degraded, but intermediates may have been formed from the reaction.

It can also be shown in Fig. 3(b) that 2-CP degradation was accompanied by SPS decomposition, such that the rapid decrease in concentration was shown at 1 min and became constant starting at 10 min. Moreover, the SPS decomposition also corresponds to the pH value and ORP, shown at Fig. 3(f), such that pH value is constantly low at approximately 3 starting at 1 min, and ORP value of approximately 500 mV. The positive ORP value for both RO water and groundwater tests are correlated to the result the SPS was not decomposed completely through the process. Moreover, it can be shown that RO water test has a high SPS decomposition compared to groundwater test. Previous research on ZVI and sulfurized ZVI has indicated that both Mg^2+^ and Ca^2+^ can cause surface corrosion.^39–42^ Groundwater tests showed higher levels of total dissolved iron compared to RO water tests. This can correspond by the ability of Mg^2+^ to dissolve surface corrosion products, while Ca^2+^ does not have this effect. Instead, Ca^2+^ ions lead to the aggregation of ZVI particles, which may reduce the surface area that is reactive. In addition, it is known that microscale ZVI exposed to SO_4_^2−^ and Cl^−^ solutions undergoes accelerated corrosion.^43–45^ The presence of Ca^2+^, Mg^2+^, SO_4_^2−^ and Cl^−^ in the groundwater may therefore contribute to increased dissolved iron levels shown at Fig. 3(d).

The ZVI-activated persulfate process demonstrated effective degradation of 2-CP under both laboratory and groundwater conditions, indicating its potential for *in situ* remediation of contaminated aquifers. However, real-world implementation may face challenges such as reagent costs (especially for persulfate), transport and mixing efficiency in heterogeneous subsurface environments, and the need to manage residual iron and sulfate. Additionally, the formation of intermediate by-products and their potential toxicity require further investigation. Despite these limitations, the process is promising due to its relatively simple operation, scalability for site-specific application, and compatibility with existing groundwater treatment infrastructure.

### 3.3. Zero valent iron characterization

**Fig. 4:**
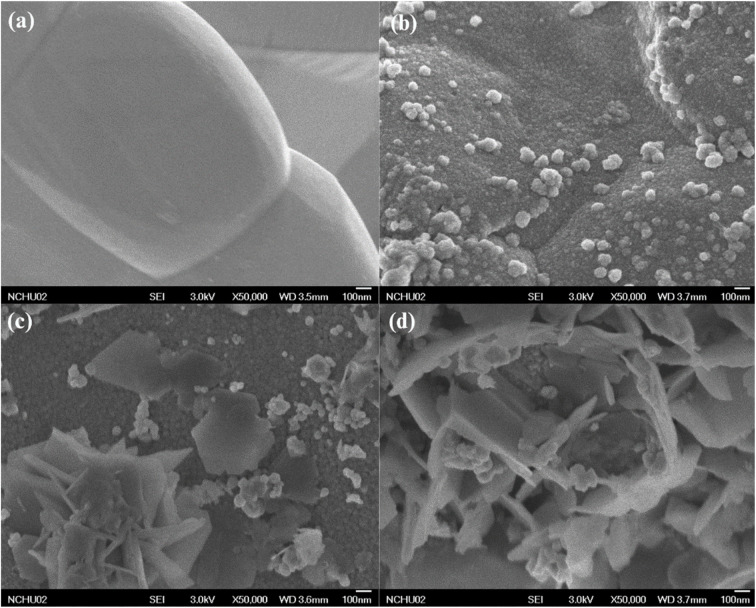
FE-SEM images of the surface of (a) unwashed ZVI, (b) acid-washed ZVI, (c) reacted ZVI in RO water, and (d) reacted ZVI in groundwater in the system of 2-CP/SPS (60 mM)/ZVI (60 mM).

Iron particles were recovered after the treatment and subsequently characterized to analyze their composition after the reaction. Fig. 4 presents the SEM images of the iron particle surfaces, highlighting changes in their morphology and surface characteristics. Fig. 4(a) and (b) illustrate the changes on the ZVI surface before and after acid washing. The original ZVI surface appears smooth, while acid washing introduces significant corrosion, resulting in increased surface roughness. This roughness likely indicates the formation of more reactive sites, produced by intense abrasion during the acid-agitated washing process. Additionally, Fig. 4(c) and (d) reveal that crystals have formed on the surface after the treatment, further indicating alterations in the surface characteristics.

**Fig. 5:**
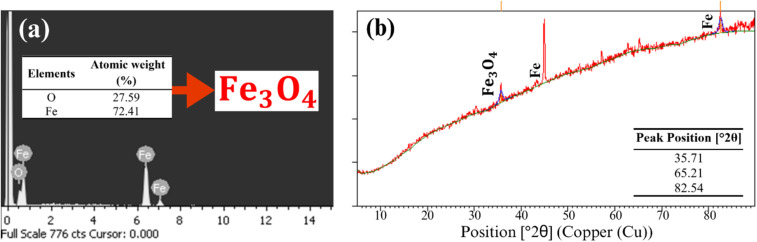
Results of (a) EDS and (b) XRD analysis for reacted ZVI in groundwater sample in the system of 2-CP/SPS (60 mM)/ZVI (60 mM).

To identify the composition of the crystal formations, EDS analysis was performed on the same iron samples. Correspondingly, only Fe was determined from both the original and acid-washed ZVI shown in Fig. S12 (ESI†). Additionally, Fig. 5(a) and (b) present compositional data from the groundwater test, identifying the presence of iron oxide magnetite (Fe_3_O_4_). This corresponds to the morphological features observed in Fig. 4(c) and (d), which show increased crystal formation on the surface of ZVI particles following oxidation. The morphology observed in Fig. 4(c) reveals the formation of acicular aggregates, resembling cryptocrystalline clusters, while Fig. 4(d) exhibits coarse, grooved aggregate structures. These features are consistent with those reported by Liang and Lai (2008), who observed similar surface morphologies on ZVI particles in a persulfate-activated ZVI system. XRD analysis was also conducted to analyze the composition of the iron particles. Shown in Fig. S13(a) and (b) (ESI†), peaks were detected at 44.97°, 65.33°, and 82.66°, which corresponds to Fe. Moreover, in Fig. 5(b), Fe peaks appeared at 65.21° and 82.54°, and Fe_3_O_4_ at 35.71°.^46^ Therefore, XRD analysis revealed that the oxidative degradation process caused formations of Fe_3_O_4_ on the iron particle surface in groundwater test. Previous study has shown that magnetite paired with ZVI resulted in high reactivity for Fenton-like process to oxidize methylene blue such that electron transfer occurred from ZVI.^47^ It has previously been found that iron oxide such as Fe_3_O_4_ may be present on ZVI surfaces and can activate SPS.^48–50^ As shown in the analyzed characteristics of the recovered iron particles after the reaction, iron oxides were determined in the particle.

## 4. Conclusion

The L9 orthogonal array, based on Taguchi's design of experiments, was employed to organize the experimental runs for 2-CP degradation, with parameters including initial pH, SPS concentration, and ZVI concentration. Analysis of the S/N ratio response identified SPS concentration as the most influential factor. The Pareto chart and *F*-test further confirmed SPS concentration's significant impact, with ANOVA results reinforcing its dominant role in 2-CP degradation. Interaction effects were observed between initial pH and SPS or ZVI concentrations. Confirmation experiments demonstrated complete 2-CP degradation, consistent with predictions. The molar ratio of SPS to ZVI influenced pH and ORP, maintaining acidic conditions (pH 2–3) and high ORP at higher SPS concentrations (2 : 1 and 4 : 1 SPS/ZVI), indicating sustained SPS activity throughout the process. Optimized conditions applied to spiked RO water and groundwater samples achieved rapid 2-CP degradation within 1 min and complete degradation in 10 min. Dissolved iron levels were higher in the groundwater test, likely due to groundwater ions (*e.g.*, Ca^2+^, Mg^2+^, SO_4_^2−^, and Cl^−^) enhancing ZVI corrosion. TOC removals were 66% and 62% for RO water and groundwater tests, respectively, indicating incomplete mineralization. Chloride quantification also suggested the formation of chlorinated intermediates, warranting further investigation to elucidate the complete degradation pathway. Future studies should focus on identifying intermediate species to better understand the reaction mechanisms and the potential formation of persistent or toxic by-products. SEM-EDS and XRD analyses identified Fe_3_O_4_ formation on ZVI surfaces. The results suggest that this optimized SPS/ZVI treatment method can effectively degrade 2-CP in natural groundwater. Its application could enhance the management of chlorophenol contamination in environmental remediation processes, providing an alternative approach to mitigate pollutants in groundwater systems.

## Conflicts of interest

There are no conflicts to declare.

## Supplementary Material

RA-015-D5RA01495F-s001

## Data Availability

The data that support the findings of this study are available on request from the corresponding author, Chenju Liang.
