# Supplementary material for: Taguchi orthogonal optimization for the oxidative degradation of 2-chlorophenol using zero valent iron-activated persulfate
Source: RSC Adv. 2025 Jul 15;15(31):25030–40. doi: 10.1039/d5ra01495f (PMC12262041; doi:10.1039/d5ra01495f)
Supplement: RA-015-D5RA01495F-s001 [file RA-015-D5RA01495F-s001.pdf]

Supplementary Information (SI) for RSC Advances.
This journal is © The Royal Society of Chemistry 2025

# Supplementary Materials

---

## Taguchi Orthogonal Optimization for the Oxidative Degradation of 2-Chlorophenol using Zero Valent Iron-Activated Persulfate

Nikka Mae M. Buenaobra[1,2], Chenju Liang[1,*], Xuyen Thi Hong Luong[1], Khyle Glainmer N. Quiton[2]

[1] Department of Environmental Engineering, National Chung Hsing University, 145 Xingda Road, South Dist., Taichung City 402202, Taiwan

[2] School of Chemical, Biological, and Materials Engineering and Sciences, Mapúa University, Philippines

*Corresponding author. Tel.: +886-4-22856610; Fax: +886-4-22856610

E-mail address: cliang@nchu.edu.tw

**Contents**


**Table S1.** Groundwater classification and value limitations of phenols and phenolic compounds.

**Table S2.** Responses for means and S/N ratios

**Table S3.** Characterization of field groundwater.

**Figure S1.** (a) Acid washing procedure on ZVI particles and (b) recovered acid and rinsing water from acid washing of ZVI until neutral pH.

**Figure S2.** (a) Schematic experimental setup and (b) actual experimental setup.

**Figure S3.** 3D full scan spectra of 2-CP using HPLC/PDA, for determination of wavelength (275 nm) and identification of retention time (5.5 min).

**Figure S4.** Example sodium persulfate concentration calibration curve using a spectrophotometry method at a wavelength of 400 nm.

**Figure S5.** Sample logarithmic chloride ion concentration calibration curve.

**Figure S6.** Ion chromatograph of standard (top) and groundwater sample (bottom) for groundwater cation determination.

**Figure S7.** Control tests for 2-CP degradation.

**Figure S8.** PARETO chart of the standardized effects for 2-chlorophenol degradation at 95% confidence level.

**Figure S9.** Interaction plot of different factors for 2-CP degradation from data means.

**Figure S10.** Normal probability plot for 2-CP degradation.

**Figure S11.** Groundwater monitoring well located in the Department of Environmental Engineering of National Chung Hsing University.

**Figure S12.** Results of EDS analysis for (a) unwashed ZVI and (b) acid-washed ZVI in groundwater in the system of 2-CP/SPS (60 mM)/ZVI (60mM)

**Figure S13.** Results of XRD analysis for (a) unwashed ZVI and (b) acid-washed ZVI in groundwater in the system of 2-CP/SPS (60 mM)/ZVI (60mM)

**Table S1.** Groundwater classification and value limitations of phenols and phenolic compounds [1]

| **Classification** | **Intended Beneficial Use** | **Concentration (mg/L)** |
|---|---|---|
| Class A | Source of Potable Water and Other Domestic Use | < 0.001 |
| Class B | Bathing and Other Primary Contact Recreation | < 0.001 |
| Class C | Irrigation, Fish Culture, Livestock Watering | 0.05 |

**Table S2.** Responses for means and S/N ratios

| **Level** | **Means** | | | **S/N ratios** | | |
|---|---|---|---|---|---|---|
| | **Initial pH** | **SPS (mM)** | **ZVI (mM)** | **Initial pH** | **SPS (mM)** | **ZVI (mM)** |
| 1 | 91.79 | 82.95 | 91.07 | 39.24 | 38.36 | 39.16 |
| 2 | 92.43 | 91.27 | 94.42 | 39.30 | 39.20 | 39.49 |
| 3 | 88.53 | 98.53 | 87.26 | 38.89 | 39.87 | 38.78 |
| Delta | 3.90 | 15.58 | 7.16 | 0.41 | 1.51 | 0.71 |
| Rank | 3 | 1 | 2 | 3 | 1 | 2 |

**Table S3.** Characterization of field groundwater

| Items | Value | Relative error range |
|---|---|---|
| pH | 6.78 | 0.01 |
| ORP (mV) | 168.17 | 1.59 |
| Dissolved Oxygen (mg/L) | 4.06 | 0.06 |
| Total Dissolved Iron (mg/L) | 0.22 | - |
| $Fe^{2+}$ (mg/L) | 0.15 | 0.01 |
| $Fe^{3+}$ (mg/L) | 0.07 | 0.01 |
| $Cl^-$ (mg/L) | 14.07 | 0.05 |
| $SO_4^{2-}$ (mg/L) | 96.26 | 1.08 |
| $F^-$ (mg/L) | 0.13 | - |
| $Na^+$ (mg/L) | 15.19 | 0.25 |
| $K^+$ (mg/L) | 1.51 | 0.01 |
| $Ca^{2+}$ (mg/L) | 70.05 | 0.32 |
| $Mg^{2+}$ (mg/L) | 14.10 | 0.03 |
| Alkalinity (mg $CaCO_3$/L) | 135.00 | - |
| Hardness (mg $CaCO_3$/L) | 236.50 | 1.50 |
| Spiked 2-CP (mM) | 1 | - |

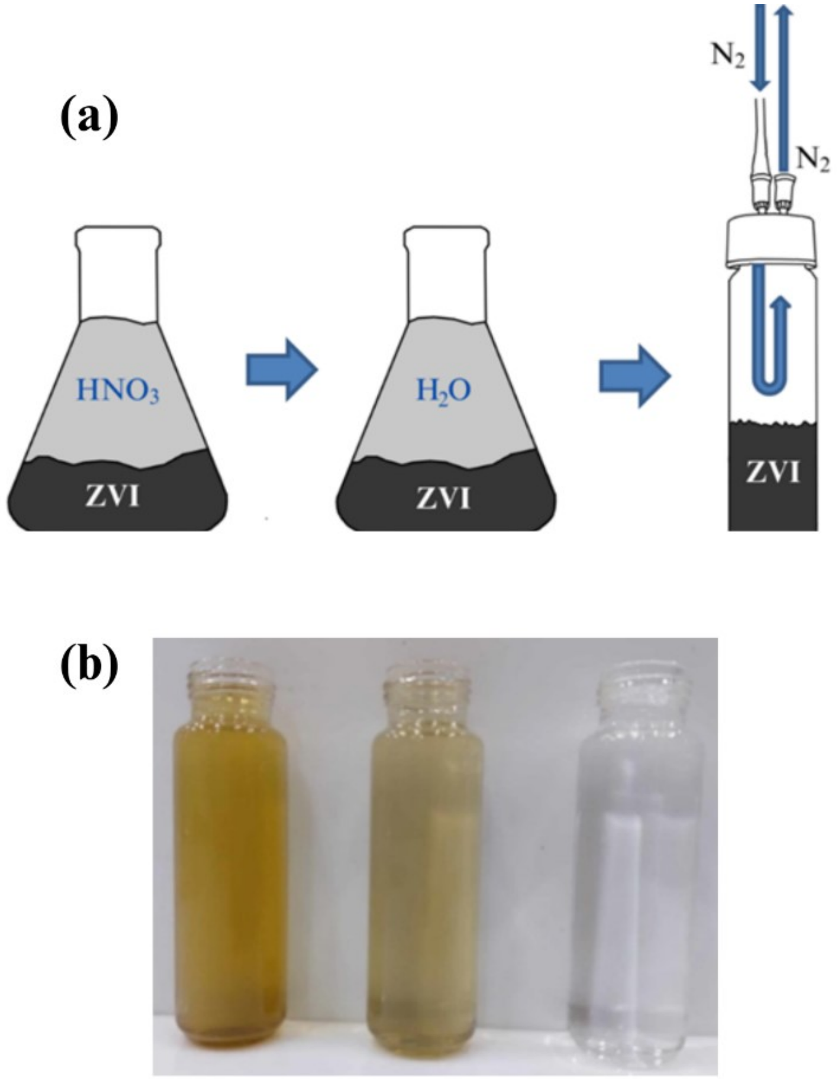


**Figure S1.** (a) Acid washing procedure on ZVI particles and (b) recovered acid and rinsing water from acid washing of ZVI until neutral pH.

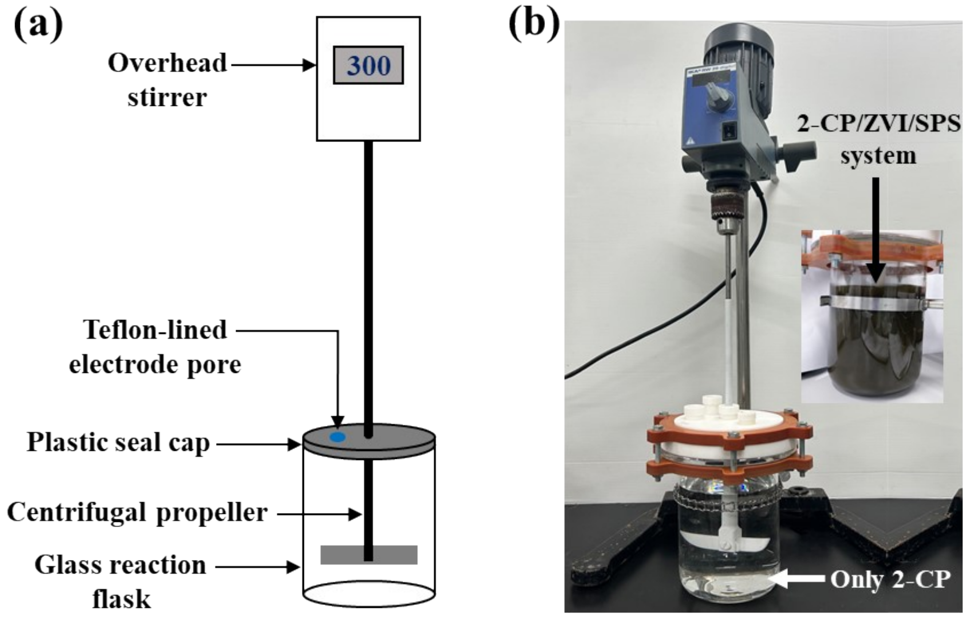


A one-liter glass reaction flask with a Teflon-seal top cover was used for the 2-CP degradation experiment. An overhead stirrer (IKA, RW 20 digital) set at 300 rpm was used during the course of reaction for mixing in the system.

**Figure S2.** (a) Schematic experimental setup and (b) actual experimental setup.

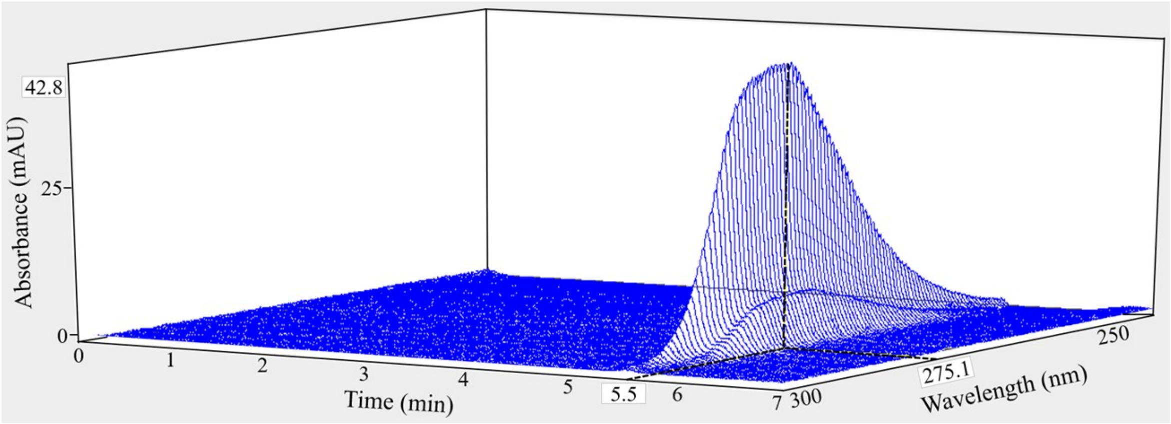


**Figure S3.** 3D full scan spectra of 2-CP using HPLC/PDA, for determination of wavelength (275 nm) and identification of retention time (5.5 min).

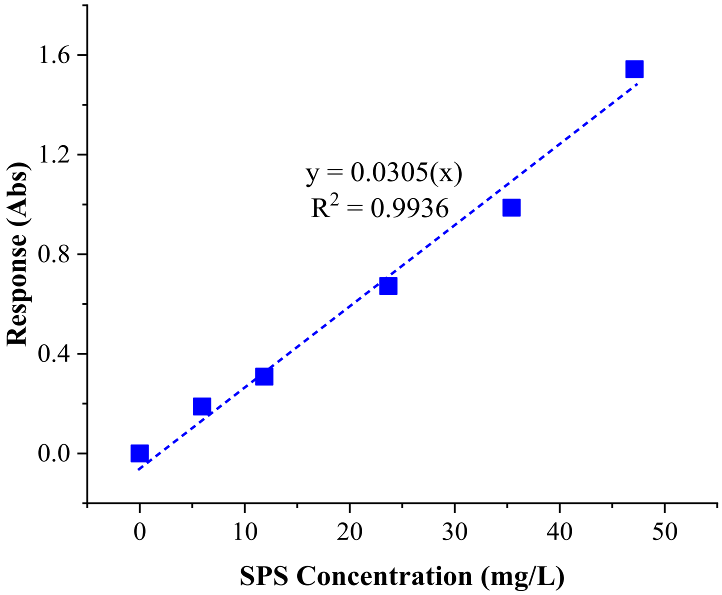


**Figure S4.** Example sodium persulfate concentration calibration curve using a spectrophotometry method at a wavelength of 400 nm.

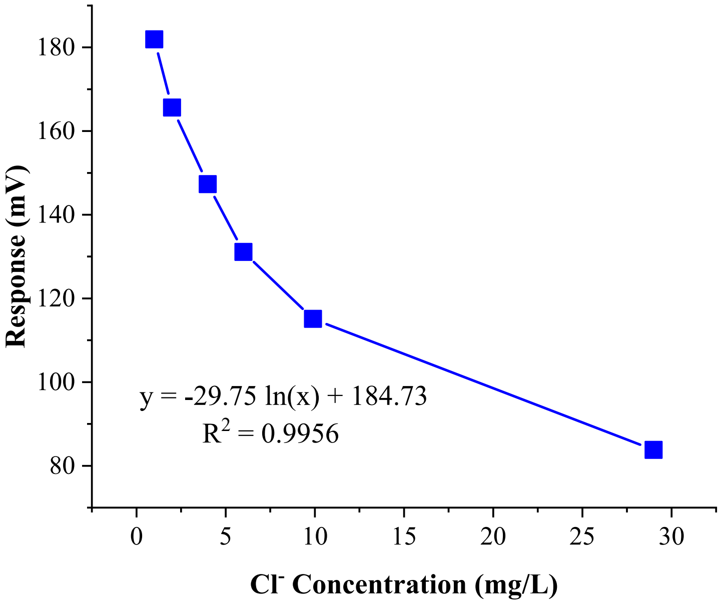


**Figure S5.** Sample logarithmic chloride ion concentration calibration curve.

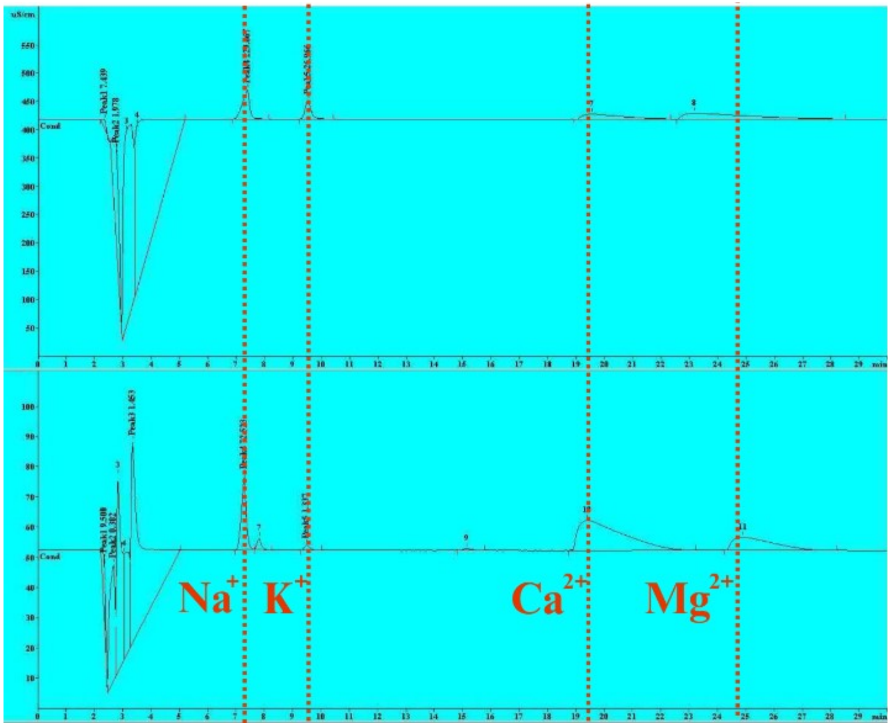


**Figure S6.** Ion chromatograph of standard (top) and groundwater sample (bottom) for groundwater cation determination.

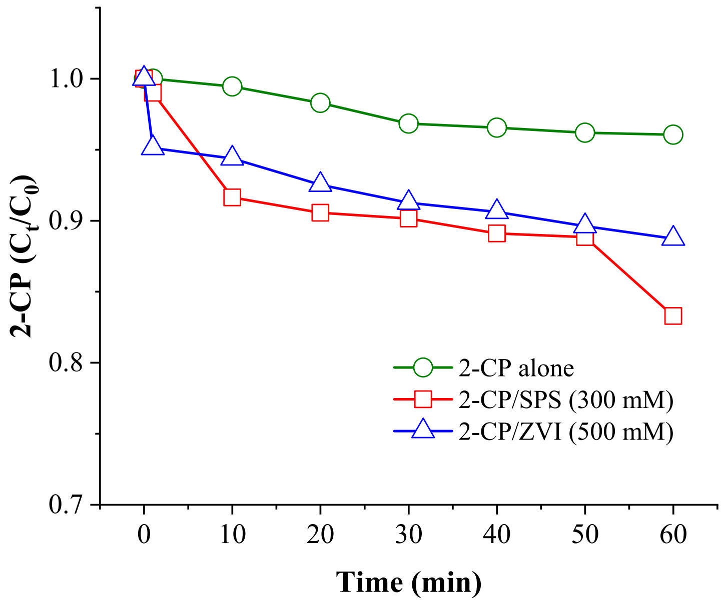


**Figure S7.** Control tests for 2-CP degradation.

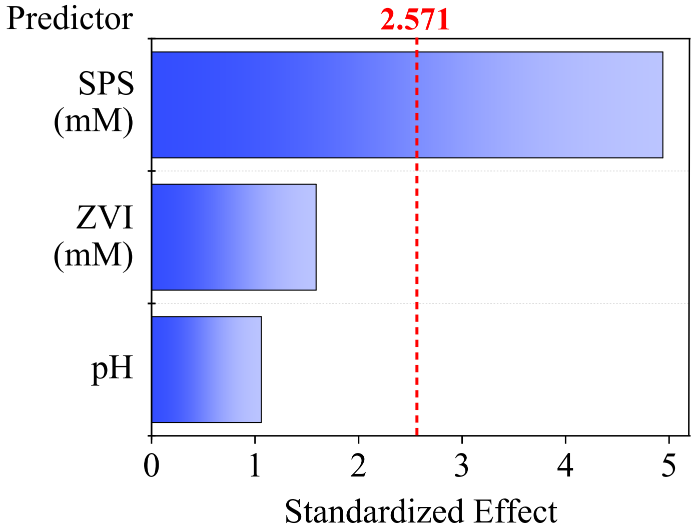

**Figure S8.** PARETO chart of the standardized effects for 2-chlorophenol degradation at

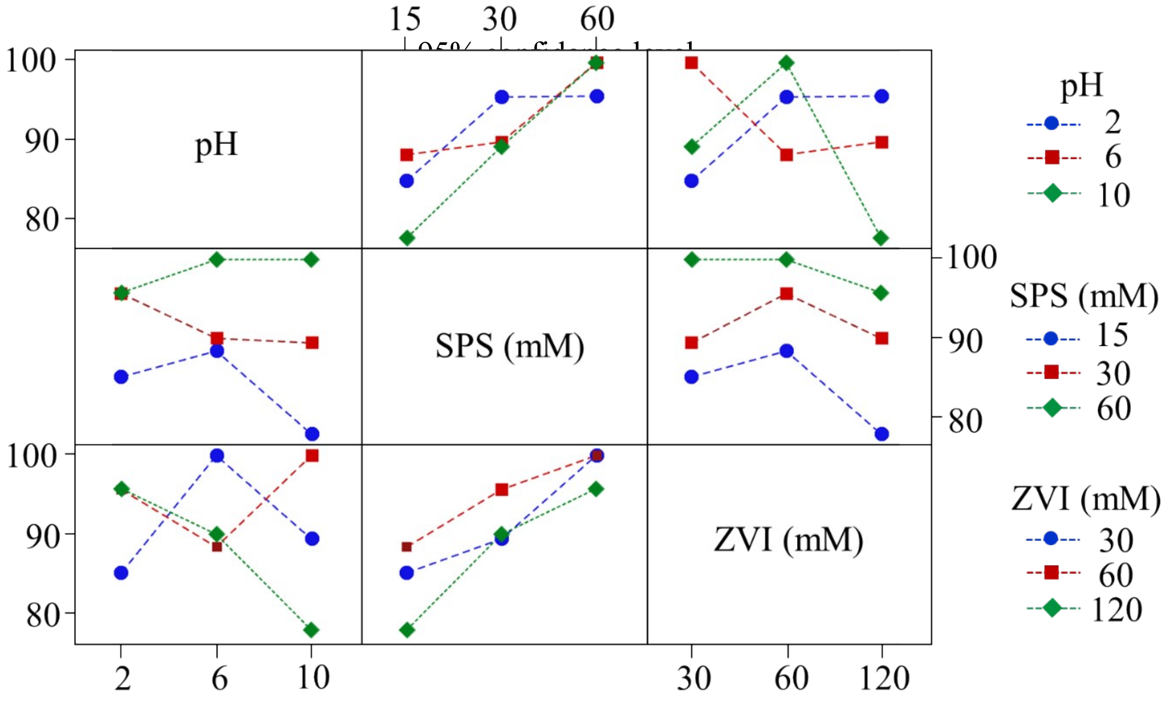


**Figure S9.** Interaction plot of different factors for 2-CP degradation from data means.

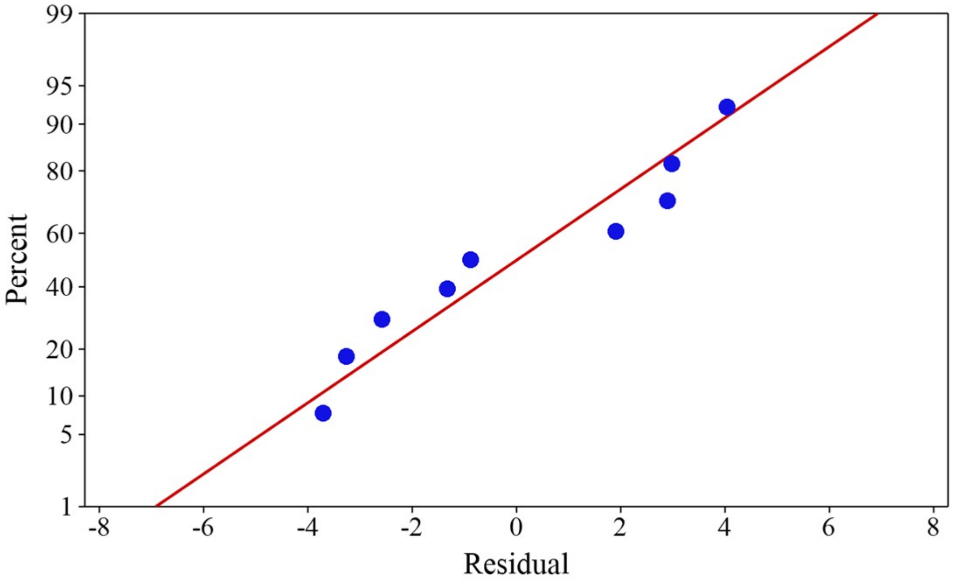


**Figure S10.** Normal probability plot for 2-CP degradation.

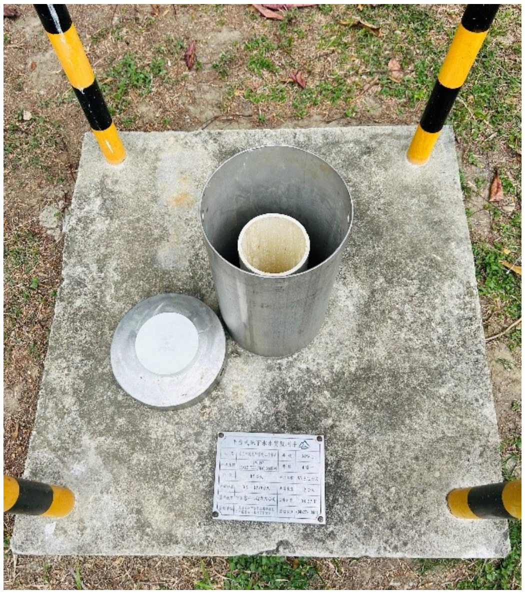

**Figure S11.** Groundwater monitoring well located in the Department of Environmental Engineering of National Chung Hsing University.

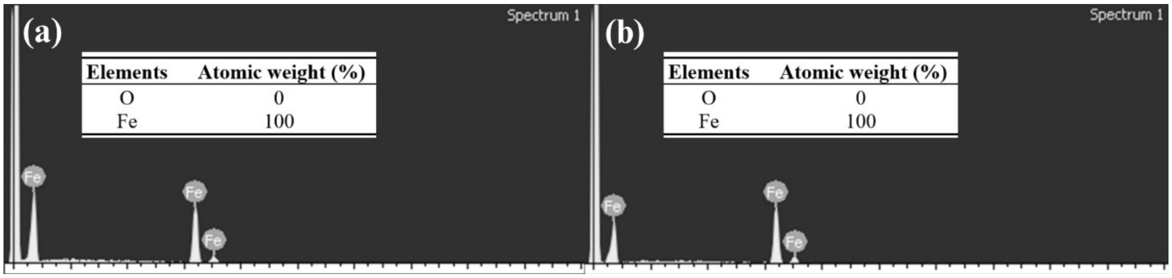


**Figure S12.** Results of EDS analysis for **(a)** unwashed ZVI and **(b)** acid-washed ZVI in groundwater in the system of 2-CP/SPS (60 mM)/ZVI (60mM)

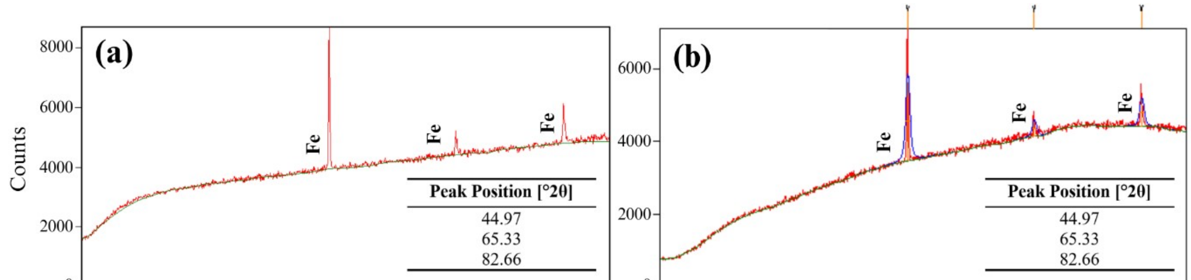

**Reference**

[1] PDENR, Water Quality Guideline and General Effluent Standards of 2016. In DENR/2016-08, Republic of the Philippines - Department of Environmental and Natural Resources (PDENR).
